# Serum Phospholipids Fatty Acids and Breast Cancer Risk by Pathological Subtype

**DOI:** 10.3390/nu12103132

**Published:** 2020-10-14

**Authors:** Virginia Lope, Ángel Guerrero-Zotano, Ana Casas, José Manuel Baena-Cañada, Begoña Bermejo, Beatriz Pérez-Gómez, Inmaculada Criado-Navarro, Silvia Antolín, Pedro Sánchez-Rovira, Manuel Ramos-Vázquez, Antonio Antón, Adela Castelló, José Ángel García-Saénz, Montserrat Muñoz, Ana de Juan, Raquel Andrés, Antonio Llombart-Cussac, Blanca Hernando, Rosa María Franquesa, Rosalia Caballero, Feliciano Priego-Capote, Miguel Martín, Marina Pollán

**Affiliations:** 1 Cancer and Environmental Epidemiology Unit, Department of Epidemiology of Chronic Diseases, National Center for Epidemiology, Carlos III Institute of Health, 28029 Madrid, Spain; bperez@isciii.es (B.P.-G.); adela.castello@uah.es (A.C.); mpollan@isciii.es (M.P.); 2 Consortium for Biomedical Research in Epidemiology & Public Health, CIBERESP, 28029 Madrid, Spain; 3 GEICAM Spanish Breast Cancer Group, 28703 Madrid, Spain; aguerrero@fivo.org (Á.G.-Z.); anacasas05@gmail.com (A.C.); josem.baena.sspa@juntadeandalucia.es (J.M.B.-C.); bego.bermejo@gmail.com (B.B.); silvia.antolin.novoa@sergas.es (S.A.); oncopsr@yahoo.es (P.S.-R.); mramosv@cog.es (M.R.-V.); aantont@gmail.com (A.A.); jagsaenz@yahoo.com (J.Á.G.-S.); mmunoz@clinic.cat (M.M.); anade.juan@scsalud.es (A.d.J.); andresraquelc@gmail.com (R.A.); allombart1@yahoo.com (A.L.-C.); bhernando@saludcastillayleon.es (B.H.); rfranquesag@chv.cat (R.M.F.); rcaballero@geicam.org (R.C.); mmartin@geicam.org (M.M.); 4 Instituto Valenciano de Oncología, 46009 Valencia, Spain; 5 Hospital Universitario Virgen Del Rocio, 41013 Sevilla, Spain; 6 Hospital Universitario Puerta del Mar, 11009 Cádiz, Spain; 7 Instituto de Investigación e Innovación Biomédica de Cádiz (INiBICA), 11009 Cádiz, Spain; 8 Hospital Clínico Universitario de Valencia, 46010 Valencia, Spain; 9 Biomedical Research Institute INCLIVA, 46010 Valencia, Spain; 10 CIBERONC, ISCIII, 28029 Madrid, Spain; 11 Analytical Chemistry Department, Universidad de Córdoba, 14071 Córdoba, Spain; inma.c.n@hotmail.es (I.C.-N.); q72prcaf@uco.es (F.P.-C.); 12 Maimónides Institute of Biomedical Research (IMIBIC), Reina Sofia University Hospital, University of Córdoba, 14071 Córdoba, Spain; 13 CIBERFES, ISCIII, 28029 Madrid, Spain; 14 Medical Oncology Unit, Complejo Hospitalario Universitario A Coruña, 15006 A Coruña, Spain; 15 Complejo Hospitalario de Jaén, 23007 Jaén, Spain; 16 Centro Oncológico de Galicia, 15009 A Coruña, Spain; 17 Hospital Universitario Miguel Servet, Instituto de Investigación Sanitaria Aragón, 50009 Zaragoza, Spain; 18 Faculty of Medicine, University of Alcalá, Alcalá de Henares, 28871 Madrid, Spain; 19 Hospital Clínico San Carlos, 28040 Madrid, Spain; 20 Medical Oncology, Hospital Clínic Barcelona, 08036 Barcelona, Spain; 21 Translational Genomics and Targeted Therapeutics, Institut d’Investigacions Biomèdiques Pi i Sunyer-IDIBAPS, 08036 Barcelona, Spain; 22 Hospital Marqués de Valdecilla, 39008 Santander, Spain; 23 Hospital Clínico Universitario Lozano Blesa, 50009 Zaragoza, Spain; 24 Hospital Arnau de Vilanova, 46015 Valencia, Spain; 25 Hospital General Yagüe, 09005 Burgos, Spain; 26 Hospital General de Vic, 08500 Barcelona, Spain; 27 Hospital General Universitario Gregorio Marañón, 28007 Madrid, Spain; 28 Instituto Investigación Sanitaria Gregorio Marañón, Universidad Complutense, 28007 Madrid, Spain

**Keywords:** breast neoplasm, breast cancer subtypes, desaturation indices, fats, EpiGEICAM

## Abstract

This study evaluates whether serum phospholipids fatty acids (PL-FAs) and markers of their endogenous metabolism are associated with breast cancer (BC) subtypes. EpiGEICAM is a Spanish multicenter matched case-control study. A lifestyle and food frequency questionnaire was completed by 1017 BC cases and healthy women pairs. Serum PL-FA percentages were measured by gas chromatography-mass spectrometry. Conditional and multinomial logistic regression models were used to quantify the association of PL-FA tertiles with BC risk, overall and by pathological subtype (luminal, HER2+ and triple negative). Stratified analyses by body mass index and menopausal status were also performed. Serum PL-FAs were measured in 795 (78%) pairs. Women with high serum levels of stearic acid (odds ratio (OR)_T3vsT1_ = 0.44; 95% confidence interval (CI) = 0.30–0.66), linoleic acid (OR_T3vsT1_ = 0.66; 95% CI = 0.49–0.90) and arachidonic to dihomo-γ-linolenic acid ratio (OR _T3vsT1_ = 0.64; 95% CI = 0.48–0.84) presented lower BC risk. Participants with high concentrations of palmitoleic acid (OR_T3vsT1_ = 1.65; 95% CI = 1.20–2.26), *trans*-ruminant palmitelaidic acid (OR_T3vsT1_ = 1.51; 95% CI = 1.12–2.02), *trans*-industrial elaidic acid (OR_T3vsT1_ = 1.52; 95% CI = 1.14–2.03), and high oleic to stearic acid ratio (OR_T3vsT1_ = 2.04; 95% CI = 1.45–2.87) showed higher risk. These associations were similar in all BC pathological subtypes. Our results emphasize the importance of analyzing fatty acids individually, as well as the desaturase activity indices.

## 1. Introduction

Breast cancer (BC) is the most frequently diagnosed cancer in women and the leading cause of cancer-related deaths worldwide [1]. In Spain, age-standardized incidence rates have increased in recent decades [2]. In 2018, it reached a rate of 101 cases per 100,000 [3], which represents 28.7% of all newly diagnosed cancers among women [1].

There is growing evidence for a plausible role of dietary factors in BC risk, but evidence for fatty acids intake is too limited to draw conclusions [4]. Several studies have shown a positive association with the intake of saturated fatty acids (SFAs) [5,6,7,8]. The relationship between monounsaturated fatty acids (MUFAs) and BC risk is more conflicting, and seems to depend on the contributing food source, such as olive oil and margarines [5,7]. On the other hand, higher consumption of marine omega-3 polyunsaturated fatty acids (n-3 PUFAs) as well as elevated intake of n-3/n-6 PUFA ratio seem to exert a protective effect on this tumor [5,7,9,10,11,12]. To assess the usual intake of fatty acids, these studies were based on commonly-used dietary questionnaires. However, self-reported methods are limited by systematic and random measurement errors [13]. Moreover, only some fatty acids, particularly those not endogenously synthesized, are good biomarkers of the diet, and are estimated by food frequency questionnaires. Therefore, the fatty acid profile in biological samples is a more accurate measure that reflects the interaction between dietary intake, de novo lipogenesis and the activity of different enzymes involved in this metabolic process [14]. Although data from a meta-analysis of prospective studies found no association between serum levels of SFAs, MUFAs and PUFAs and risk of BC [15], more recent studies have described positive associations with circulating levels of several SFAs [16,17], palmitoleic acid [18], some *trans* fatty acids [17,18,19] and the ratio of palmitoleic acid to palmitic acid (DI_16_) [18], as well as an inverse association with n-3 PUFAs among overweight/obese women [17].

BC represents a heterogeneous disease, characterized by the presence versus absence of specific receptors on the tumor cells: hormonal receptors (HR) (estrogen receptors (ER) and progesterone receptors (PR)) and human epidermal growth factor receptor 2 (HER2) [20]. It has been described that the expression of fatty acid metabolism proteins differs with respect to intrinsic molecular BC subtype [21]. To date, very few studies have analyzed the association between circulating fatty acids and BC risk defined by hormonal receptor status [16,18], and, to our knowledge, none of them have studied this relationship taking into account the HER2 status. The objective of the present study was to investigate the association between fatty acids assessed in serum phospholipids and BC risk, as well as to evaluate whether this association differed according to specific pathological BC subtype.

## 2. Materials and Methods

### 2.1. Study Population

Data came from a multicenter case-control study on female BC, including incident cases and individually matched healthy controls. Between 2006 and 2011, 1017 incident cases of BC were recruited in the Oncology departments of 23 hospitals belonging to the Spanish Breast Cancer Research Group (GEICAM) (https://www.geicam.org/). These hospitals are located in nine of the 17 Spanish Autonomous Regions, where 78% of the Spanish population resides. The oncologists invited the patients to participate at the time of diagnosis. Participants were between 18 and 70 years old, with no history of BC. They should reside in the area attended by the hospital, and they should be able to answer the epidemiological questionnaire. Each patient was matched with a healthy control of a similar age (±5 years), generally selected by the patient. This could be a friend, a neighbor or a work colleague residing in the same city. Of the 2494 women who were initially invited to participate in the EpiGEICAM study (1362 patients and 1132 controls), 1017 case–control pairs participated; thus, the overall participation rate was 82% (75% in BC cases and 90% in controls).

BC cases were classified according to the following pathological subtypes based on local pathology reports [22]: HR positive tumors (HR+: ER+ or PR+ with HER2-); HER2 positive tumors (HER2+ irrespective of ER or PR status); and triple negative tumors (TN: ER-, PR- and HER2-). ER, PR and HER2 positivity were defined according to ASCO/CAP guidelines [23,24].

During the first three months after diagnosis, patients and controls filled a structured questionnaire collecting anthropometric and demographic data, personal and family background, gynecological, obstetric and occupational history, smoking status, physical activity and diet. Postmenopausal status was based on self-reported information and was defined as absence of menstruation in the past 12 months. Dietary intake during the preceding five years was estimated using a 117-item semi-quantitative food frequency questionnaire (FFQ), similar to the Harvard questionnaire [25], and adapted to and validated in different Spanish adult populations [26]. The responses for each food item were converted to mean daily intake (in grams) and total energy intake (in kcals/day). Verification and data entry were carried out at GEICAM headquarters. The accuracy of the information recorded in the database was verified randomly by selecting and reviewing 10% of the questionnaires. Additionally, a basal serum sample was collected from patients and healthy controls for phospholipids fatty acid (PL-FA) analysis.

All subjects gave their informed consent for inclusion before participating in the study. This was conducted in accordance with the Declaration of Helsinki, and the protocol was approved on 25 May 2006 by the Ethics Committee of Hospital Clínico San Carlos (EpiGEICAM-01, Committee internal code E-06/141). Patient information was anonymized and de-identified prior to analysis. Further details regarding the study design have been previously published [27,28].

### 2.2. Analysis of Serum PL-FAs

PL-FAs were determined by using the protocol proposed by Criado-Navarro et al. [29], which is based on the isolation of PLs using 30 mg HybridSPE^®^ cartridges from Supelco (Bellefonte, PA, USA), derivatization of the resulting extract to convert PL-FAs into their more volatile fatty acid methyl esters of phospholipids (PL-FAMEs) and gas chromatography coupled to mass spectrometry (GC–MS) analysis. The NIST Mass Spectral Search Program v.11.0 (NIST, Washington, DC, USA) was used for spectral search (Mainlib and Replib libraries). Tentative identification was reported when the correlation between experimental and database spectra was above 0.75 in normal search mode. Confirmatory analysis was carried out by analysis of a FAMEs multistandard from Sigma–Aldrich (Steinheim, Germany).

The relative concentration of each PL-FA, expressed as percentage of total PL-FAs in serum, was quantified by integrating the area under the peak and dividing the result by the total PL-FA area. The variability of the determination, expressed as the variation coefficient in percentages, ranged from 0.3 to 14.9%. We obtained the relative levels of 25 individual PL-FAs, expressed as the percent of total serum PL-FAs, including: SFAs (14:0, 15:0, 16:0, 17:0, 18:0, 20:0 and 22:0), *cis*-MUFAs (16:1 n-7, 17:1, 18:1 n-9, 20:1 n-9 and 22:1 n-9), n-3 PUFAs (18:3, 20:5 and 22:6), n-6 PUFAs (18:2, 18:3, 20:2, 20:3, 20:4 and 22:2), ruminant *trans* fatty acids (16:1 n-7 and 18:1 n-7) and industrial *trans* fatty acids (18:1 n-9 and 18:2 n-6). We also analyzed the ratio of 16:1 n-7 palmitoleic acid to 16:0 palmitic acid (SCD-16 or DI_16_) and the ratio of 18:1 n-9 oleic acid to 18:0 stearic acid (SCD-18 or DI_18_), as biomarkers of the stearoyl-CoA desaturase 1 (SCD-1) (Δ9-desaturase) expression [30]; the ratio of 20:4 n-6 arachidonic acid to 20:3 n-6 dihomo-γ-linolenic acid, indicator of the fatty acid desaturase 1 (FADS1) activity (Δ5-desaturase), and the ratio of 20:3 n-6 dihomo-γ-linolenic acid to 18:2 n-6 linoleic acid, indicator of the activity of Δ6-desaturase and elongase [31].

### 2.3. Statistical Methods

Serum PL-FA levels were analyzed for 795 case-control participant pairs. Given the matching nature of our data, missing values were imputed using Multiple Imputation by Chained Equation [32]. This methodology was used to fill incomplete information for height (9.2%), weight (2.8%), waist circumference (2.6%), age at menarche (0.8%), age at first birth (4.6%), hormone replacement therapy (HRT) use (4.9%), smoking (0.4%), alcohol consumption (2.9%), overall physical activity during the previous year (8.5%) and caloric intake (4.4%). Imputation models also included the following potential explanatory variables with complete data: case–control status, age, parity, menopausal status, educational level, hypercholesterolemia, previous benign breast problems, family history of BC and all serum PL-FAs. Five imputed data sets were generated using the multiple imputation procedure implemented in STATA [33]. A complete BC case analysis was also performed to check the validity of the imputation.

Descriptive characteristics of participants were summarized for patients and controls. We calculated absolute figures and percentages for categorical variables and means and standard deviations for continuous variables. Significant differences between patients and controls were tested using Pearson chi-square for categorical and Student’s t-test for continuous variables.

To analyze the association between BC and relative percentage of serum PL-FAs, the latter were divided into tertiles, based on the distribution of serum levels in controls. Second and third tertiles were compared with the first tertile (reference) using conditional logistic regression models adjusted for educational level (no formal school or first grade; second grade or vocational training; university graduate), body mass index (BMI) one year prior to the interview, menopausal status (these two variables were included in the models with the corresponding interaction term), age at menarche (continuous), age at first birth (<20, 20–24; 25–29; ≥30; nulliparous), HRT use (never; ever), alcohol consumption (g/day), self-assessed physical activity during the previous year (sedentary or slightly active; moderately active; active or very active), history of benign breast disease (no; yes), family history of BC (none; second degree; first degree) and caloric intake (Kcal/day). The linear trend across tertiles was also tested with the Wald test. In addition to categorical analyses, each group of serum PL-FAs was also modelled through a restricted quadratic spline with knots at the 5th, 50th and 95th percentiles [34]. These restricted quadratic splines allowed two different quadratic trends on either side of the median PL-FAs that were restricted to be linear below the 5th percentile and above the 95th percentile; thus, they could reproduce a large variety of smooth dose-response curves while avoiding implausible shapes at extreme PL-FA levels. As sensitivity analyses, we conducted stratified analyses by BMI and menopausal status, comparing the third tertile of each PL-FA with the first tertile. The potential effect modification was evaluated including in the models the corresponding interaction terms. To take into account the problem of multiple testing, *p*-values were suitably adjusted using the false discovery rate as proposed by Benjamini and Hochberg [35].

Finally, multinomial logistic regression models were used to evaluate the association of serum PL-FAs with each of the aforementioned intrinsic BC subtypes. These models were adjusted for age (continuous), recruiting hospital and the same set of potential confounders described above. Heterogeneity of effects was tested using the Wald test, comparing the coefficients obtained for the different cancer subtypes. All statistical analyses were performed with STATA/MP 15.1 software (StataCorp LLC, College Station, TX, USA).

## 3. Results

Table 1 shows the main characteristics of the 795 case–control pairs with information on the relative concentrations of serum PL-FAs. Their mean age was 50 years. Compared with controls, patients had lower educational level, higher proportion of premenopausal women and postmenopausal overweight women, higher caloric intake, higher frequency of previous benign breast diseases and more relatives with BC.

Table 2 shows mean percent of each serum PL-FA (percent of total) in patients and controls, as well as BC risk by tertiles of PL-FAs. When we compared women in the third tertile with women in the first tertile, those with high relative concentrations of stearic acid (odds ratio (OR)_T3vsT1_ = 0.44; 95% confidence interval (CI) = 0.30–0.66) and linoleic acid (OR_T3vsT1_ = 0.66; 95% CI = 0.49–0.90) presented lower BC risk, while women with high levels of palmitoleic acid showed higher risk (OR_T3vsT1_ = 1.65; 95% CI = 1.20–2.26). Associations were also observed with other PL-FAs whose relative serum concentrations were much lower. In this way, we found a protective association with behenic acid, as well as a positive association linked to pentadecanoic, gondoic and γ-linolenic acids. On the other hand, women with high relative concentrations of *trans* fatty acids showed a significant increase in BC risk, both of animal origin (OR_T3vsT1_ = 1.44; 95% CI = 1.07–1.93)—mainly due to the action of palmitelaidic acid—and of industrial origin (OR_T3vsT1_ = 1.38; 95% CI = 1.04–1.85)—mainly due to the action of elaidic acid. Finally, regarding desaturation indices, the ratio of oleic acid to stearic acid (SCD-18) (OR_T3vsT1_ = 2.04; 95% CI = 1.45–2.87) and the ratio between dihomo-γ-linolenic and linoleic acids (OR_T3vsT1_ = 1.35; 95% CI = 1.02–1.78) were positively associated with BC risk, while the ratio between arachidonic and dihomo-γ-linolenic acids showed and inverse relationship (OR_T3vsT1_ = 0.64; 95% CI = 0.48–0.84).

When these analyses were repeated, stratifying by menopausal status and BMI (Table 3), no significant heterogeneity was observed in the associations. However, we observed that the association of *trans* fatty acids of animal origin was particularly intense in postmenopausal women (OR_T3vsT1_ = 1.87; 95% CI = 1.20–2.92), while the BC risk associated with those of industrial origin was higher in premenopausal women (OR_T3vsT1_ = 1.67; 95% CI = 1.14–2.47). An excess risk of BC associated with elevated serum *cis*-MUFAs levels was observed only in postmenopausal women and in overweight/obese women. In the latter women, there was also an increased risk associated with *trans* ruminant fatty acids (OR_T3vsT1_ = 1.73; 95% CI = 1.14–2.64), a stronger inverse association linked to the FADS1 desaturation index (OR_T3vsT1_ = 0.52; 95% CI = 0.35–0.77), and a protective relationship that was not observed in women with BMI < 25 Kg/m^2^, linked to docosahexaenoic acid (DHA) (OR_T3vsT1_ = 0.71; 95%CI = 0.47–1.06).

In general, the association between PL-FAs and BC risk by pathological subtype was similar to that observed for total BC risk (Table 4), although the associations with HER2+ tumors and TN tumors failed to attain statistical significance, probably due to the smaller number of cases. In particular, high relative concentrations of the saturated pentadecanoic fatty acid were associated with increased risk of HR+ and HER2+ tumors, but with a decreased risk of TN tumors (*p* for heterogeneity = 0.098). On the other hand, serum erucic acid concentrations were inversely associated with HR + tumors and, to a lesser extent, with TN tumors, while a positive association with HER2 + tumors was observed (*p* for heterogeneity = 0.030).

**Figure 1:**
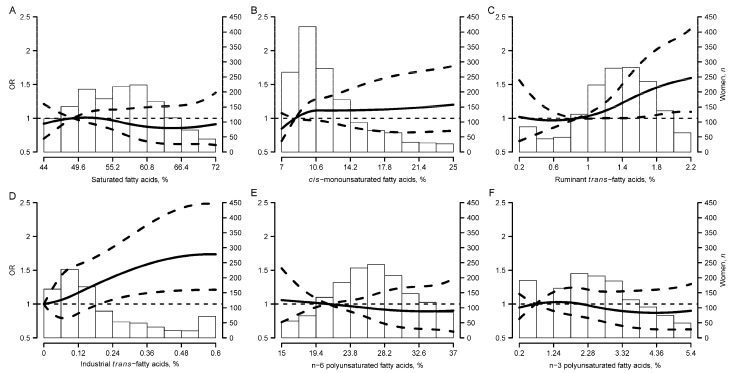
Breast cancer risk as a smooth function of serum phospholipid fatty acids. Curves represent adjusted odds ratios (solid lines) and 95% confidence intervals (dashed lines) based on restricted quadratic splines for (**A**) saturated fatty acids, (**B**) *cis-*monounsaturated fatty acids, (**C**) ruminant *trans*−fatty acids, (**D**) industrial *trans*−fatty acids, (**E**) n-6 polyunsaturated fatty acids and (**F**) n-3 polyunsaturated fatty acids, with knots at their 5th, 50th and 95th percentiles. The reference value for each group of fatty acids was set at the median of the first tertile (56.4%, 10.9%, 1.3%, 0.1%, 26.6% and 2.4% respectively). Odds ratios were obtained from conditional logistic regression models adjusted for educational level, body mass index one year prior to the interview, menopausal status, age at menarche, age at first birth, hormone replacement therapy use, alcohol consumption, self-assessed physical activity during the previous year, history of benign breast disease, family history of breast cancer and caloric intake. Bars represent the histogram of each group of fatty acids.

Figure 1 depicts the dose-response curve for the different groups of fatty acids. As can be seen, BC risk increases as the relative concentrations of *trans* fatty acids increase. A slight increasing trend is also observed with *cis*-MUFA levels and a slight decreasing trend is associated with n-6 PUFAs.

## 4. Discussion

In the present study, we found evidence that higher relative concentrations of phospholipid monounsaturated palmitoleic and gondoic acids, *trans* fatty acids and a high SCD-18 desaturation index were associated with increased BC risk, while increased concentrations of stearic acid, linoleic acid and FADS1 index were inversely associated. This relationship was similar in all BC pathological subtypes.

Stearic acid is a long-chain saturated fatty acid found primarily in foods typical of a Western diet, including meat, poultry, fish, grain products, chocolate and milk fats [36,37]. The protective association provided by this fatty acid has been previously reported in premenopausal women by Saadatian-Elahi et al. in a meta-analysis of fatty acids in biological samples and BC risk [38]. The influence of stearic acid on the inhibition of tumor cells in vitro and tumor development in vivo has been demonstrated for many years [39]. More recently, stearate has been shown to induce apoptosis of human BC cells involving protein kinase C in the signaling cascade [40] and, in addition, to reduce visceral adipose tissue by apoptosis of preadipocytes [37].

Palmitoleic acid is an n-7 MUFA biosynthesized from palmitic acid by the action of the SCD-1 enzyme in the liver. Although not commonly found in food, it is present in macadamia nuts, blue-green algae and marine oils [41,42]. Elevated plasma palmitoleic acid levels have been associated with increased risk of general [18,43,44] and post-menopausal BC [38]. The SCD-1 (or Δ9-desaturase) is an enzyme that converts SFAs into MUFAs in the liver. Several studies have involved the expression and activity of this enzyme in the development and progression of various types of cancer, including BC [30,45]. Thus, some epidemiological studies have shown that high serum concentrations of oleic acid together with low levels of stearic acid (high SCD-18 desaturation index) [46,47], as well as high levels of palmitoleic acid to the detriment of palmitic acid concentrations (high SCD-16 index) [18,43,44]—both indicators of high SCD-1 activity—are associated with an increased BC risk. Although we have not detected differences by histological type, it has been described that SCD-1 expression is greater in HR+ and HER2+ BC subtypes than in TN breast tumors [48].

Linoleic acid, the essential fatty acid of the n-6 PUFA family, is found in typical foods of the Western diet, such as vegetable oils, nuts, seeds, meat and eggs [49]. The role of linoleic acid in cancer development remains unclear. A systematic review of randomized controlled trials concluded that there is no evidence to show that the addition of linoleic acid to the diet increases the concentration of inflammatory markers [50]. In relation to BC, Zhou et al., in a meta-analysis of 12 prospective studies, showed that both linoleic acid intake and serum levels of this fatty acid were associated with decreased BC risk, although none of the associations was statistically significant [49]. This inverse relationship, and in line with what we observed in our results, was more evident among premenopausal women than among postmenopausal women.

Since our results show an inverse association with linoleic acid, a borderline positive association with serum levels of dihomo-γ-linolenic acid, and no relationship with arachidonic acid, the FADS2 desaturation index (indicator of Δ6-desaturase activity) was positively related to BC, while the FADS1 index (indicator of Δ5-desaturase expression) showed an inverse association. Although an increased Δ6-desaturase activity has been found in human BC tissue [51], these enzymes do not appear to influence the development of BC [31]. Only two previous studies have observed an association with this tumor in the opposite direction to our results [52,53].

Regarding *trans* fatty acids, a recent meta-analysis indicated that while dietary intake of these fatty acids was not associated with BC risk, a significant positive association was observed with serum *trans* fats in postmenopausal women [19]. We have detected an increased BC risk associated with high serum concentrations of *trans*-palmitelaidic and elaidic acids. These are precisely the two *trans* fatty acids that Chajes et al. found to be positively associated with this tumor in the E3N-EPIC cohort [43]. Palmitelaidic acid is a natural *trans* fatty acid produced by microbial hydrogenation of palmitoleic acid in the rumen of ruminants and, consequently, is present in all fats of these animals [54], although it can also be produced endogenously by the partial β oxidation of dietary vaccenic acid [55]. Although studies evaluating the relationship between BC and levels of this *trans* fatty acid in biomarkers are scarce, positive associations have been described in the serum of current postmenopausal smokers [56], in the erythrocytes membrane of overweight/obese women from the Nurses’ Health Study II [17], and in the plasma of a cohort of Australian women, although in the latter case the association was only found with ER-/PR- tumors [16]. Elaidic acid, the *trans* form of oleic acid, is the main industrial *trans* fatty acid that is produced during the partial hydrogenation of vegetable oils, and is a reliable biomarker of highly processed foods among European populations [57]. Chajes et al., in a study that evaluated the variation in the percent of weight change at five years, found that doubling the plasma concentration of elaidic acid was associated with a decreased risk of weight loss and an increased risk of weight gain during the five-year follow-up in women [58]. Other studies, although they have not been able to separate *trans* fatty acids of industrial origin into their different individual isomers, have described a positive association between this type of *trans* fatty acids and BC risk in general [17], and ER- tumors in particular [18]. In contrast, elaidic acid was not associated with BC risk in another prospective Italian study [47].

There are several possible biological hypotheses by which *trans* fatty acids could influence the development of BC. These fatty acids are positively associated with markers of inflammation [59], which may be related to breast carcinogenesis [4]. Other possible mechanisms proposed are the elevation of circulating estrogen levels, induced oxidative stress, and body weight regulation [19].

Despite these results, the mean content of *trans* fatty acids in food products in Spain is low, and has been declining since 2010 [60]. Although Spanish legislation does not yet regulate its content, in May 2019 a new regulation of the European Commission came into force limiting the content of *trans* fats of industrial origin in food to a maximum of 2 g per 100 g of fat [61], regulation that has a transitional period of two years so that industries can adapt. However, this regulation only regulates *trans* fats of industrial origin and, precisely, the group of foods with the highest content of *trans* fatty acids in Spain are dairy products, which contain fatty acids of natural origin [60].

One of the main strengths of this study is that histologically confirmed patients and controls were recruited in nine Spanish regions located throughout the Spanish geography, which allowed us to have a broad representation of fatty acid intake in Spain. To our knowledge, this is the first study exploring the association between serum PL-FAs and BC risk considering HER2 status separately. The large number of premenopausal participants allowed us to adequately explore the association of serum PL-FAs with BC risk in these women. Another important strength is the wide range of fatty acids measured in serum phospholipids. In addition, this biomarker, compared to traditional self-reported assessment methods, constitutes a more objective measure of the bioavailable amount of fatty acids. Finally, The GC-MS analysis represents a powerful platform for the analysis of fatty acids in complex samples with exceptional sensitivity [62].

Several limitations should also be addressed. First, the study relied on self-reported data, and recall bias may be present in some exposures. However, we believe that this misclassification is most likely non-differential. Second, the fact of having selected friends as controls of BC cases could imply greater similarity in lifestyles, including eating habits and fat intake. However, this possible bias would imply an underestimation of the effects studied and, therefore, the associations reported here would underestimate the real ones. Third, although most of the established risk factors were taken into account, residual unmeasured confounders associated with serum PL-FA levels, such as cholesterol, triglycerides, insulin or other dietary factors, may have interfered with the detected associations. Fourth, chance findings could be occurred due to the large number of tests performed. However, to control for multiple comparisons, the false discovery rate (q values) was calculated using the Benjamini and Hochberg correction, a powerful test but at the cost of a higher number of Type I errors. Fifth, PL-FA levels are expressed as a percentage of total fatty acids, reflecting relative concentrations. Therefore, changes in one PL-FA affect the relative concentration of the others, particularly those representing lower proportions. Sixth, statistical power was limited in the analyses by menopausal status, by BMI and by pathological subtypes, since TN tumors are relatively uncommon in Spanish population [63]. Seventh, it is possible that metabolic alterations prior to BC diagnosis could have contributed to altering serum PL-FA levels in our patients. However, we believe that this is unlikely, since this effect would be observed mainly in the more advanced stages of the disease, and we have not found differences by pathological subtype. Eighth, our study is based on a single baseline measurement for blood levels, thus, we have not been able to evaluate changes over time. Finally, although there are other biological markers, such as adipose tissue, more appropriate to reflect long-term dietary intake [64], the fatty acid composition of serum phospholipids can be a useful tool in epidemiological studies [65], and it is considered a convenient alternative for the medium- to long-term assessment of habitual availability and metabolism of fatty acids in large-scale epidemiological studies [66].

## 5. Conclusions

In conclusion, our results suggest that SFAs, MUFAs and PUFAs cannot be considered as homogeneous groups when studying their association with BC risk, but should be analyzed individually. Furthermore, serum PL-FA levels are not only a reflection of what we eat, but are also the result of the activity of different enzymes involved in a variety of metabolic processes. Our results suggest that higher intake of stearic and linoleic acids, as well as a reduction in the consumption of palmitoleic acid and *trans* fatty acids, could be a good strategy for BC prevention. Additional studies are needed to better understand the influence of PL-FAs on BC development.

## Figures and Tables

**Table 1:** Baseline characteristics among breast cancer cases and controls.

Characteristics	Cases (*n* = 795)		Controls (*n* = 795)		*p*-Value
Age, mean (SD)	50.5	(9.5)	50.3	(9.4)	0.730 ^a^
Educational level, *n* (%)					
No formal school education/First grade	173	(22)	132	(17)	0.001 ^a^
Second grade/Vocational training	424	(53)	406	(51)	
University graduate	198	(25)	257	(32)	
Age at menarche, mean (SD)	12.6	(1.5)	12.5	(1.5)	0.159 ^b^
Age at first birth, mean (SD)	26.5	(4.4)	26.2	(4.4)	0.129 ^b^
Number of children, *n* (%)					
None	172	(22)	178	(22)	0.970 ^a^
1–2	479	(60)	479	(60)	
3–4	133	(17)	128	(16)	
>4	11	(1)	10	(1)	
Menopausal status, *n* (%)					
Premenopausal	451	(57)	412	(52)	0.050 ^a^
Postmenopausal	344	(43)	383	(48)	
Body mass index, Kg/m^2^, mean (SD)					
Premenopausal	24.5	(4.3)	24.8	(4.5)	0.146 ^b^
Postmenopausal	27.1	(4.9)	26.3	(4.1)	0.009 ^b^
Waist circumference (cm)	88.1	(14.2)	87.2	(12.8)	0.146 ^b^
Hormone replacement therapy use, *n* (%)					
Never	702	(88)	712	(90)	0.386 ^b^
Ever	93	(12)	83	(10)	
Previous benign breast problems, *n* (%)					
No	620	(78)	651	(82)	0.052 ^a^
Yes	175	(22)	144	(18)	
Family history of breast cancer, *n* (%)					
None	597	(75)	639	(80)	0.033 ^a^
Second degree	101	(13)	85	(11)	
First degree	97	(12)	71	(9)	
Self-assessed physical activity last year, *n* (%)					
Sedentary/slightly active	297	(37)	266	(33)	0.332 ^b^
Moderately active	281	(35)	313	(39)	
Active/very active	217	(27)	217	(27)	
Alcohol consumption (g/day), *n* (%)					
No	190	(24)	184	(23)	0.537 ^b^
<10	440	(55)	469	(59)	
≥10	164	(21)	142	(18)	
Smoking, *n* (%)					
Never smoker	343	(43)	332	(42)	0.367 ^b^
Former smoker ≥ 6 months ago	208	(26)	203	(26)	
Smoker or former smoker < 6 months	244	(31)	260	(33)	
Caloric intake (Kcal/day), mean (SD)	1991.1	(623.4)	1903.8	(659.9)	0.005 ^b^
Tumor subtype ^c^, *n* (%)					
HR+	532	(67)			
HER2+	162	(20)			
TN	99	(12)			

Abbreviations: HR+ = hormone receptor positive tumors (ER+ and/or PR+, with HER2-); HER2+ = human epidermal growth factor receptor 2 positive tumors; TN = triple negative tumors. ^a^ *p*-value resulting from Pearson Chi-Square test (completed variables). ^b^ *p*-value resulting from conditional logistic regression models (imputed variables). ^c^ Two breast cancer cases could not be classified.

**Table 2:** Breast cancer risk by tertiles of serum phospholipid fatty acids.

	Cases		Controls		Tertile 2			Tertile 3				
	Mean %	SD	Mean %	SD	OR ^a^	(95% CI)	*p*-Value	OR ^a^	(95% CI)	*p*-Value	*p*-Trend	*q*-Trend ^b^
**SFAs**												
14:0 myristic acid	0.42	(0.31)	0.40	(0.28)	0.90	(0.69–1.19)	0.464	0.96	(0.72–1.28)	0.783	0.797	0.797
15:0 pentadecanoic acid	0.19	(0.17)	0.18	(0.16)	1.15	(0.88–1.51)	0.308	1.37	(1.03–1.82)	0.032	0.031	0.088
16:0 palmitic acid	39.78	(8.73)	38.87	(8.21)	0.87	(0.65–1.14)	0.308	1.23	(0.91–1.66)	0.181	0.199	0.388
17:0 margaric acid	0.27	(0.54)	0.24	(0.15)	1.14	(0.86–1.52)	0.349	1.25	(0.92–1.69)	0.150	0.150	0.326
18:0 stearic acid	15.90	(4.77)	16.76	(5.02)	0.97	(0.72–1.32)	0.865	0.44	(0.30–0.66)	<0.001	<0.001	0.009
20:0 arachidic acid	0.09	(0.32)	0.10	(0.30)	1.34	(0.95–1.89)	0.096	1.05	(0.78–1.41)	0.743	0.646	0.771
22:0 behenic acid	0.01	(0.05)	0.01	(0.06)	0.43	(0.30–0.61)	<0.001	0.70	(0.51–0.96)	0.025	0.017	0.061
Total SFAs	56.67	(7.72)	56.57	(7.86)	0.84	(0.62–1.12)	0.231	0.86	(0.64–1.16)	0.319	0.340	0.503
***cis*-MUFAs**												
16:1 n-7 palmitoleic acid	0.32	(0.30)	0.29	(0.26)	1.48	(1.10–1.97)	0.008	1.65	(1.20–2.26)	0.002	0.002	0.015
17:1 heptadecenoic acid	0.03	(0.03)	0.03	(0.03)	0.80	(0.61–1.06)	0.120	1.04	(0.77–1.40)	0.810	0.792	0.797
18:1 n-9 oleic acid	12.10	(5.54)	11.92	(5.36)	1.06	(0.82–1.38)	0.658	1.17	(0.86–1.60)	0.306	0.313	0.483
20:1 n-9 gondoic acid	0.12	(0.54)	0.08	(0.10)	1.20	(0.88–1.64)	0.243	1.68	(1.23–2.30)	0.001	0.001	0.009
22:1 n-9 erucic acid	0.12	(0.42)	0.22	(1.23)	0.99	(0.72–1.36)	0.932	0.77	(0.57–1.02)	0.071	0.077	0.178
Total cis-MUFAs	12.69	(5.61)	12.54	(5.54)	1.06	(0.81–1.38)	0.680	1.19	(0.87–1.62)	0.273	0.282	0.454
***cis-*n-6 PUFAs**												
18:2 linoleic acid	17.37	(4.30)	17.65	(4.08)	0.91	(0.69–1.18)	0.468	0.66	(0.49–0.90)	0.009	0.010	0.053
18:3 γ-linolenic acid	0.04	(0.42)	0.02	(0.06)	0.87	(0.63–1.19)	0.371	1.39	(1.06–1.82)	0.019	0.018	0.061
20:2 eicosadienoic acid	0.11	(0.15)	0.10	(0.13)	0.92	(0.68–1.25)	0.608	1.18	(0.85–1.62)	0.322	0.274	0.454
20:3 dihomo-γ-linolenic acid	1.99	(1.20)	1.85	(1.02)	1.05	(0.81–1.38)	0.695	1.30	(0.99–1.71)	0.063	0.060	0.148
20:4 arachidonic acid	7.05	(2.61)	7.15	(2.63)	1.02	(0.77–1.33)	0.909	0.94	(0.71–1.25)	0.689	0.698	0.783
22:2 docosadienoic acid	0.02	(0.03)	0.02	(0.03)	0.87	(0.65–1.17)	0.364	1.17	(0.89–1.55)	0.255	0.249	0.454
Total cis-n-6 PUFAs	26.59	(5.97)	26.80	(5.96)	0.99	(0.76–1.30)	0.960	0.87	(0.64–1.19)	0.391	0.415	0.591
***cis*-n-3 PUFAs**												
18:3 α-linolenic acid	0.06	(0.06)	0.06	(0.07)	0.96	(0.71–1.31)	0.807	1.04	(0.76–1.44)	0.794	0.767	0.797
20:5 eicosapentaenoic acid (EPA)	0.46	(0.51)	0.50	(0.58)	1.02	(0.77–1.33)	0.905	0.93	(0.69–1.26)	0.648	0.642	0.771
22:6 docosahexaenoic acid (DHA)	1.94	(1.30)	1.98	(1.24)	0.92	(0.70–1.21)	0.541	0.90	(0.67–1.20)	0.468	0.465	0.637
Total cis-n-3 PUFAs	2.46	(1.64)	2.54	(1.65)	1.03	(0.78–1.36)	0.836	0.95	(0.70–1.28)	0.747	0.746	0.797
**Total trans Fatty acids**												
16:1 n-7t palmitelaidic acid	0.24	(0.19)	0.22	(0.18)	1.51	(1.12–2.04)	0.006	1.51	(1.12–2.02)	0.007	0.012	0.056
18:1 n-9t elaidic acid	0.21	(0.66)	0.25	(1.89)	1.53	(1.14–2.04)	0.005	1.52	(1.14–2.03)	0.004	0.005	0.031
18:1 n-7t vaccenic acid	1.11	(0.67)	1.07	(0.46)	1.06	(0.80–1.41)	0.663	1.18	(0.88–1.57)	0.268	0.268	0.454
18:2 n-6t linolelaidic acid	0.01	(0.04)	0.01	(0.12)	0.85	(0.60–1.21)	0.379	0.92	(0.69–1.21)	0.540	0.559	0.713
Ruminant trans-fatty acids ^c^	1.36	(0.69)	1.29	(0.50)	1.38	(1.05–1.81)	0.021	1.44	(1.07–1.93)	0.016	0.017	0.061
Industrial trans-fatty acids ^d^	0.22	(0.66)	0.26	(1.89)	1.14	(0.86–1.49)	0.359	1.38	(1.04–1.85)	0.028	0.028	0.086
Ratio cis-PUFA n-6/n-3 ^e^	13.74	(2.20)	13.33	(2.25)	1.08	(0.82–1.42)	0.595	1.09	(0.81–1.47)	0.551	0.553	0.713
**Desaturation indices**												
SCD-16: 16:1n-7c/16:0 ^e^	0.01	(1.93)	0.01	(1.97)	1.40	(1.06–1.87)	0.020	1.25	(0.91–1.72)	0.161	0.160	0.329
SCD-18: 18:1n-9/18:0 ^e^	0.73	(1.58)	0.69	(1.63)	2.21	(1.58–3.08)	<0.001	2.04	(1.45–2.87)	<0.001	<0.001	0.009
FADS1: 20:4n-6/20:3n-6 ^e^	3.74	(1.54)	4.01	(1.48)	0.79	(0.61–1.02)	0.067	0.64	(0.48–0.84)	0.001	0.001	0.009
FADS2: 20:3n-6/18:2n-6 ^e^	0.10	(1.02)	0.10	(1.75)	1.12	(0.86–1.47)	0.401	1.35	(1.02–1.78)	0.034	0.034	0.090

Abbreviations: CI, confidence interval; FADS, fatty acid desaturase; MUFAs, monounsaturated fatty acids; n-3 PUFAs, omega-3 polyunsaturated fatty acids; n-6 PUFAs, omega-6 polyunsaturated fatty acids; OR, odds ratio; SCD, stearoyl-CoA desaturase; SFAs, saturated fatty acids. ^a^ Adjusted for educational level, body mass index, menopausal status, age at menarche, age at first birth (with a category of history of nulliparous), hormone replacement therapy use, alcohol consumption, physical activity, previous benign breast problems, family history of breast cancer and energy intake. Adjusted for age and hospital by design. ^b^ *p*-value for linear trend in tertiles following the Benjamini and Hochberg procedure. ^c^ Includes 16:1 n-7t and 18:1 n-7t. ^d^ Includes 18:1 n-9t and 18:2t. ^e^ Geometric mean and geometric standard deviation.

**Table 3:** Breast cancer risk for the third tertile compared with the first tertile of each serum phospholipid fatty acid by menopausal status and by body mass index.

	Menopausal Status							Body Mass Index						
	Premenopausal			Postmenopausal				BMI < 25 Kg/m^2^			BMI ≥ 25 Kg/m^2^			
	OR ^a^	95% CI	*p*-Value	OR ^a^	95% CI	*p*-Value	*p*-het ^b^	OR ^a^	95% CI	*p*-Value	OR ^a^	95% CI	*p*-Value	*p*-het ^b^
**SFAs**														
14:0 myristic acid	1.15	(0.79–1.68)	0.452	0.77	(0.51–1.17)	0.219	0.147	0.80	(0.54–1.18)	0.262	1.18	(0.79–1.76)	0.422	0.143
15:0 pentadecanoic acid	1.36	(0.94–1.97)	0.102	1.38	(0.90–2.12)	0.140	0.977	1.25	(0.85–1.83)	0.261	1.50	(0.98–2.30)	0.060	0.476
16:0 palmitic acid	1.13	(0.76–1.69)	0.543	1.34	(0.87–2.06)	0.179	0.603	1.24	(0.82–1.85)	0.307	1.18	(0.79–1.77)	0.415	0.889
17:0 margaric acid	1.24	(0.84–1.83)	0.270	1.25	(0.81–1.95)	0.314	0.980	1.28	(0.86–1.89)	0.221	1.22	(0.81–1.85)	0.339	0.884
18:0 stearic acid	0.49	(0.30–0.81)	0.005	0.39	(0.23–0.66)	<0.001	0.353	0.50	(0.31–0.82)	0.005	0.38	(0.23–0.63)	<0.001	0.277
20:0 arachidic acid	1.11	(0.74–1.65)	0.624	1.00	(0.67–1.50)	0.981	0.687	0.88	(0.60–1.30)	0.527	1.25	(0.84–1.85)	0.265	0.183
22:0 behenic acid	0.74	(0.49–1.10)	0.137	0.65	(0.42–1.02)	0.062	0.791	0.68	(0.46–1.01)	0.057	0.73	(0.49–1.09)	0.120	0.740
Total SFAs	0.88	(0.59–1.30)	0.513	0.84	(0.56–1.27)	0.415	0.852	0.73	(0.49–1.09)	0.124	1.03	(0.68–1.54)	0.902	0.230
***cis*-MUFAs**														
16:1 n-7 palmitoleic acid	1.43	(0.95–2.15)	0.084	1.96	(1.24–3.08)	0.004	0.350	1.44	(0.95–2.19)	0.084	1.89	(1.24–2.89)	0.003	0.374
17:1 heptadecenoic acid	1.11	(0.74–1.65)	0.620	0.96	(0.63–1.46)	0.840	0.686	1.07	(0.71–1.60)	0.759	1.01	(0.68–1.51)	0.944	0.911
18:1 n-9 oleic acid	1.14	(0.75–1.73)	0.547	1.22	(0.80–1.87)	0.355	0.760	1.07	(0.71–1.60)	0.758	1.32	(0.87–2.02)	0.196	0.446
20:1 n-9 gondoic acid	1.87	(1.24–2.82)	0.003	1.49	(0.96–2.33)	0.078	0.584	1.77	(1.17–2.66)	0.007	1.60	(1.05–2.45)	0.029	0.745
22:1 n-9 erucic acid	0.72	(0.49–1.05)	0.086	0.83	(0.55–1.26)	0.387	0.589	0.84	(0.57–1.23)	0.359	0.70	(0.48–1.03)	0.073	0.431
Total cis-MUFAs	1.00	(0.65–1.53)	0.997	1.47	(0.95–2.25)	0.081	0.151	0.99	(0.66–1.49)	0.954	1.46	(0.96–2.23)	0.080	0.167
***cis-*n-6 PUFAs**														
18:2 linoleic acid	0.59	(0.39–0.88)	0.009	0.85	(0.54–1.34)	0.478	0.201	0.70	(0.47–1.05)	0.082	0.63	(0.41–0.98)	0.040	0.774
18:3 γ-linolenic acid	1.47	(1.02–2.11)	0.036	1.29	(0.86–1.92)	0.216	0.587	1.49	(1.02–2.17)	0.037	1.30	(0.91–1.87)	0.148	0.577
20:2 eicosadienoic acid	1.11	(0.73–1.68)	0.636	1.27	(0.81–1.98)	0.296	0.652	1.26	(0.84–1.90)	0.265	1.09	(0.71–1.67)	0.709	0.555
20:3 dihomo-γ-linolenic acid	1.31	(0.91–1.89)	0.153	1.29	(0.86–1.93)	0.212	0.971	1.28	(0.87–1.87)	0.209	1.33	(0.90–1.96)	0.152	0.844
20:4 arachidonic acid	0.87	(0.60–1.25)	0.446	1.05	(0.70–1.58)	0.818	0.500	1.15	(0.77–1.72)	0.506	0.80	(0.55–1.17)	0.254	0.185
22:2 docosadienoic acid	1.23	(0.85–1.78)	0.264	1.11	(0.75–1.65)	0.603	0.735	1.13	(0.77–1.66)	0.529	1.21	(0.84–1.76)	0.308	0.774
Total cis-n-6 PUFAs	0.83	(0.55–1.24)	0.353	0.94	(0.61–1.46)	0.793	0.645	1.02	(0.67–1.53)	0.943	0.74	(0.48–1.13)	0.160	0.260
***cis*-n-3 PUFAs**														
18:3 α-linolenic acid	1.01	(0.66–1.56)	0.951	1.07	(0.68–1.68)	0.777	0.933	1.01	(0.66–1.54)	0.979	1.09	(0.71–1.67)	0.705	0.806
20:5 eicosapentaenoic acid (EPA)	1.03	(0.68–1.56)	0.873	0.88	(0.57–1.36)	0.576	0.526	0.89	(0.60–1.33)	0.578	0.99	(0.65–1.50)	0.953	0.682
22:6 docosahexaenoic acid (DHA)	0.91	(0.61–1.35)	0.642	0.88	(0.59–1.32)	0.548	0.913	1.13	(0.76–1.69)	0.536	0.71	(0.47–1.06)	0.090	0.090
Total cis-n-3 PUFAs	1.00	(0.66–1.49)	0.985	0.91	(0.60–1.39)	0.669	0.752	1.12	(0.75–1.68)	0.575	0.81	(0.54–1.21)	0.302	0.250
**Total trans fatty acids**														
16:1 n-7t palmitelaidic acid	1.36	(0.93–2.00)	0.115	1.66	(1.07–2.56)	0.023	0.527	1.33	(0.90–1.96)	0.151	1.73	(1.13–2.64)	0.011	0.287
18:1 n-9t elaidic acid	1.75	(1.20–2.55)	0.004	1.29	(0.86–1.93)	0.213	0.238	1.72	(1.16–2.56)	0.007	1.34	(0.91–1.96)	0.133	0.340
18:1 n-7t vaccenic acid	1.02	(0.70–1.49)	0.910	1.42	(0.93–2.17)	0.109	0.247	1.17	(0.79–1.72)	0.430	1.20	(0.80–1.81)	0.375	0.937
18:2 n-6t linolelaidic acid	1.06	(0.73–1.54)	0.757	0.78	(0.51–1.18)	0.235	0.314	1.07	(0.74–1.55)	0.719	0.78	(0.54–1.14)	0.196	0.244
Ruminant trans-fatty acids ^c^	1.19	(0.82–1.73)	0.361	1.87	(1.20–2.92)	0.006	0.114	1.22	(0.82–1.81)	0.330	1.73	(1.14–2.64)	0.010	0.196
Industrial trans-fatty acids ^d^	1.67	(1.14–2.47)	0.009	1.14	(0.77–1.71)	0.508	0.169	1.67	(1.10–2.54)	0.016	1.17	(0.79–1.74)	0.439	0.280
Ratio cis-PUFA n-6/n-3	1.06	(0.71–1.59)	0.778	1.08	(0.71–1.64)	0.711	0.961	1.02	(0.68–1.53)	0.939	1.17	(0.79–1.75)	0.428	0.601
**Desaturation indices**																
SCD-16: 16:1n-7c/16:0	1.08	(0.71–1.63)	0.723	1.48	(0.95–2.31)	0.087	0.392	1.11	(0.74–1.69)	0.610	1.38	(0.91–2.11)	0.131	0.466
SCD-18: 18:1n-9/18:0	2.04	(1.29–3.21)	0.002	2.05	(1.28–3.26)	0.003	0.972	1.79	(1.14–2.81)	0.011	2.34	(1.48–3.70)	<0.001	0.250
FADS1: 20:4n-6/20:3n-6	0.68	(0.47–0.98)	0.040	0.59	(0.39–0.88)	0.009	0.567	0.78	(0.54–1.12)	0.178	0.52	(0.35–0.77)	0.001	0.130
FADS2: 20:3n-6/18:2n-6	1.23	(0.85–1.79)	0.273	1.49	(1.00–2.22)	0.050	0.478	1.32	(0.90–1.93)	0.152	1.35	(0.91–2.01)	0.138	0.871

Abbreviations: BMI, body mass index; CI, confidence interval; FADS, fatty acid desaturase; MUFAs, monounsaturated fatty acids; n-3 PUFAs, omega-3 polyunsaturated fatty acids; n-6 PUFAs, omega-6 polyunsaturated fatty acids; OR, odds ratio; SCD, stearoyl-CoA desaturase; SFAs, saturated fatty acids. ^a^ Adjusted for educational level, body mass index, menopausal status (these two variables were included in the models with the corresponding interaction term), age at menarche, age at first birth (with a category of history of nulliparous), hormone replacement therapy use, alcohol consumption, physical activity, previous benign breast problems, family history of breast cancer and energy intake. Adjusted for age and hospital by design. ^b^ *p*-value for heterogeneity. ^c^ Includes 16:1 n-7t and 18:1 n-7t. ^d^ Includes 18:1 n-9t and 18:2t.

**Table 4:** Breast cancer risk for the third tertile compared with the first tertile of each serum phospholipid fatty acid by pathological subtype*.

	HR+ (*n* = 532)			HER2+ (*n* = 162)			TN (*n* = 99)			
	OR ^a^	95% CI	*p*-Value	OR ^a^	95% CI	*p*-Value	OR ^a^	95% CI	*p*-Value	*p*-Het ^b^
**SFAs**										
14:0 myristic acid	1.10	(0.83–1.45)	0.502	0.87	(0.57–1.33)	0.526	0.72	(0.43–1.19)	0.200	0.208
15:0 pentadecanoic acid	1.44	(1.09–1.91)	0.010	1.24	(0.81–1.90)	0.330	0.81	(0.48–1.35)	0.421	0.098
16:0 palmitic acid	1.18	(0.90–1.55)	0.229	1.18	(0.78–1.81)	0.434	0.84	(0.50–1.42)	0.516	0.445
17:0 margaric acid	1.20	(0.91–1.59)	0.197	1.16	(0.75–1.81)	0.505	1.39	(0.81–2.37)	0.232	0.854
18:0 stearic acid	0.68	(0.51–0.91)	0.009	0.68	(0.43–1.07)	0.094	0.80	(0.46–1.38)	0.416	0.847
20:0 arachidic acid	1.01	(0.78–1.31)	0.945	1.18	(0.79–1.77)	0.417	1.08	(0.66–1.76)	0.772	0.755
22:0 behenic acid	0.81	(0.63–1.05)	0.113	0.92	(0.62–1.35)	0.656	0.94	(0.58–1.50)	0.782	0.757
Total SFAs	1.00	(0.76–1.32)	0.986	0.85	(0.56–1.29)	0.448	0.89	(0.53–1.49)	0.647	0.721
***cis*-MUFAs**										
16:1 n-7 palmitoleic acid	1.41	(1.06–1.88)	0.017	1.59	(1.02–2.47)	0.041	1.28	(0.74–2.24)	0.377	0.809
17:1 heptadecenoic acid	1.01	(0.76–1.32)	0.967	1.04	(0.69–1.58)	0.838	1.22	(0.73–2.06)	0.445	0.767
18:1 n-9 oleic acid	1.14	(0.86–1.51)	0.353	0.89	(0.58–1.37)	0.596	1.18	(0.70–1.98)	0.538	0.516
20:1 n-9 gondoic acid	1.35	(1.02–1.79)	0.034	1.72	(1.11–2.66)	0.015	1.56	(0.91–2.65)	0.103	0.547
22:1 n-9 erucic acid	0.70	(0.54–0.91)	0.009	1.21	(0.82–1.79)	0.330	0.86	(0.53–1.42)	0.564	0.030
Total cis-MUFAs	1.12	(0.85–1.49)	0.417	0.94	(0.61–1.46)	0.786	1.21	(0.72–2.02)	0.477	0.683
***cis-*n-6 PUFAs**										
18:2 linoleic acid	0.68	(0.51–0.90)	0.008	0.80	(0.52–1.25)	0.328	0.91	(0.54–1.54)	0.725	0.486
18:3 γ-linolenic acid	1.23	(0.96–1.58)	0.104	1.16	(0.79–1.71)	0.457	1.79	(1.12–2.87)	0.016	0.264
20:2 eicosadienoic acid	0.99	(0.75–1.30)	0.946	1.43	(0.94–2.18)	0.097	1.31	(0.77–2.24)	0.320	0.197
20:3 dihomo-γ-linolenic acid	1.18	(0.89–1.56)	0.258	1.26	(0.84–1.90)	0.263	1.30	(0.76–2.22)	0.340	0.905
20:4 arachidonic acid	0.87	(0.66–1.15)	0.316	0.84	(0.55–1.29)	0.428	1.07	(0.63–1.84)	0.792	0.715
22:2 docosadienoic acid	1.14	(0.87–1.48)	0.341	1.24	(0.83–1.85)	0.287	1.02	(0.61–1.69)	0.948	0.803
Total cis-n-6 PUFAs	0.84	(0.64–1.12)	0.232	0.95	(0.62–1.46)	0.811	1.08	(0.63–1.84)	0.781	0.635
***cis*-n-3 PUFAs**										
18:3 α-linolenic acid	0.97	(0.73–1.28)	0.833	1.29	(0.83–1.99)	0.253	1.19	(0.70–2.02)	0.526	0.408
20:5 eicosapentaenoic acid (EPA)	0.86	(0.65–1.15)	0.302	1.09	(0.70–1.68)	0.712	0.94	(0.55–1.59)	0.805	0.600
22:6 docosahexaenoic acid (DHA)	0.89	(0.68–1.17)	0.417	0.96	(0.62–1.49)	0.849	0.77	(0.45–1.30)	0.322	0.786
Total cis-n-3 PUFAs	0.95	(0.72–1.25)	0.707	0.99	(0.64–1.53)	0.973	0.79	(0.45–1.36)	0.388	0.764
**Total Trans Fatty Acids**										
16:1 n-7t palmitelaidic acid	1.35	(1.01–1.79)	0.040	1.52	(0.96–2.39)	0.071	1.47	(0.86–2.50)	0.160	0.861
18:1 n-9t elaidic acid	1.31	(1.01–1.71)	0.044	1.33	(0.87–2.03)	0.187	1.37	(0.83–2.26)	0.214	0.985
18:1 n-7t vaccenic acid	1.14	(0.87–1.51)	0.342	1.02	(0.65–1.57)	0.946	0.99	(0.59–1.65)	0.957	0.783
18:2 n-6t linolelaidic acid	0.89	(0.69–1.14)	0.354	0.96	(0.65–1.42)	0.848	1.32	(0.83–2.08)	0.239	0.258
Ruminant trans-fatty acids ^c^	1.25	(0.94–1.66)	0.131	1.46	(0.92–2.32)	0.107	1.14	(0.68–1.90)	0.618	0.725
Industrial trans-fatty acids ^d^	1.23	(0.93–1.62)	0.153	1.37	(0.87–2.14)	0.175	1.27	(0.76–2.14)	0.367	0.899
Ratio cis-PUFA n-6/n-3	1.07	(0.81–1.42)	0.631	0.94	(0.61–1.44)	0.768	1.31	(0.76–2.26)	0.340	0.605
**Desaturation Indexes**										
SCD-16: 16:1n-7c/16:0	1.22	(0.92–1.63)	0.171	1.29	(0.83–2.02)	0.263	1.02	(0.59–1.76)	0.942	0.770
SCD-18: 18:1n-9/18:0	1.54	(1.15–2.06)	0.004	1.23	(0.79–1.91)	0.355	1.38	(0.76–2.50)	0.291	0.626
FADS1: 20:4n-6/20:3n-6	0.66	(0.50–0.87)	0.004	0.55	(0.35–0.87)	0.010	0.66	(0.39–1.12)	0.122	0.746
FADS2: 20:3n-6/18:2n-6	1.24	(0.94–1.65)	0.134	1.44	(0.93–2.23)	0.100	1.22	(0.72–2.06)	0.466	0.797

Abbreviations: HR+, hormone receptor positive tumors (ER+ and/or PR+, with HER2-); HER2+, human epidermal growth factor receptor 2 positive tumors; TN, triple negative tumors; CI, confidence interval; FADS, fatty acid desaturase; MUFAs, monounsaturated fatty acids; n-3 PUFAs, omega-3 polyunsaturated fatty acids; n-6 PUFAs, omega-6 polyunsaturated fatty acids; OR, odds ratio; SCD, stearoyl-CoA desaturase; SFAs, saturated fatty acids. * Two breast cancer cases could not be classified. ^a^ Adjusted for age, hospital, educational level, body mass index, menopausal status, age at menarche, age at first birth (with a category of history of nulliparous), hormone replacement therapy use, alcohol consumption, physical activity, previous benign breast problems, family history of breast cancer and energy intake. ^b^ *p*-value for heterogeneity. ^c^ Includes 16:1 n-7t and 18:1 n-7t. ^d^ Includes 18:1 n-9t and 18:2t.
